# Human endogenous retroviruses and ageing

**DOI:** 10.1186/s12979-021-00228-x

**Published:** 2021-03-26

**Authors:** Mikko Hurme, Graham Pawelec

**Affiliations:** 1 Faculty of Medicine and Health Technology, Tampere University, Tampere, Finland; 2 Department of Immunology, University of Tübingen, Tübingen, Germany; 3 Health Sciences North Research Institute, Sudbury, Ontario Canada

Over evolutionary time, the human genome has incorporated large amounts of genetic material from ancient retroviral infections, comprising ca. 8% of the total DNA. The majority of human endogenous retroviruses (HERVs) are defective due to the accumulation of mutations and deletions but some proviruses, especially in the youngest families (e.g. HERV-K (HML-2), contain intact genes permitting the production of proviral proteins (for a review, see [1]). The functions of HERVs have been under active investigation for decades and it is now known that far from being quiescent, some are involved in several crucial physiologic processes, clearly advantageous or essential to the host, e.g. placentation, neuroprotection and differentiation of cells in early embryos. However, there are now many publications also showing that HERVs are involved in the pathogenesis of several diseases (autoimmune, inflammatory, malignancies) [1].

The regulation of the expression of HERVs is very complex. In healthy individuals their activation is controlled by epigenetic mechanisms, but some constitutive expression is observed in several organs of the body. It is obvious that genetic location of the provirus is decisive in the regulation of its expression, i.e. provirus insertions can be within or nearby a gene, resulting in a complex network of inductive signals. Therefore, studies demonstrating associations between HERV expression and a given disease condition would be more informative if the location of the provirus, and its surroundings, would be analyzed. One obvious pathogenetic mechanism would naturally be the production of new infective viruses, which would then insert into the genome as a provirus and modify the expression of the surrounding genes (insertional mutagenesis). Thus far, however, there is no evidence that this mechanism would be functional in humans. In addition to these complexities in the regulation of the transcription of HERVs, it is now known that the end products, i.e. the *env*, *gag*, *pol*-encoded proteins, are directly involved in the pathogenesis of some diseases, especially those of an autoimmune nature [2]. It seems that these proteins (especially env) can be recognized as foreign and are able to induce the production of autoantibodies (the shared epitope model). Also in this case it is probable that the various proviruses would have a different effect (i.e. the protein should be intact enough to be able to activate antibody production, but should also express the shared epitope).

There is now evidence that the aging-associated changes in the defense mechanisms of the body modify the response against HERVs (eg. immunosenescence). For example, the activity of the HERV-K (HML-2) provirus at 1q22, which is relatively intact, is increased in older adults, and its expression is associated with a neutrophil-dominated inflammatory response [3]. As transcriptional changes in this response were very similar to those observed in influenza infection, in this respect it seems that this HERV behaves like a normal virus. Now, in a pre-print (not yet peer-reviewed) [4], Liu et al. have addressed the issue of the role of HERVs in cellular senescence, which is of major importance in ageing and inflammageing. Using various in vitro senescence models with cells from healthy individuals or from progeroid syndrome patients they observed high expression of several retroelements, including the HERV-K (HML-2) family retroviruses. This expression was probably due to the loss of epigenetic control (i.e. the same mechanism regulating the expression of HERVs in early embryonic cells). This expression also led to the production of HERV-K (HML-2) env protein. To examine the possible functions of the HERVs produced in the culture supernatants of senescent cells, they added this to cultures of “younger” cells and, surprisingly, a more rapid induction of senescence was observed. Moreover, treatment of the culture supernatant with anti-Env antibodies abolished this senescence-enhancing effect. Thus, they concluded that the HERV-Env protein is a major player in senescence induction in these in vitro models. Should it transpire that is also has a role in vivo in cell types the ageing of which resembles that observed in the in vitro senescence models, a new avenue for understanding and manipulating ageing processes in humans may be opened up.

## Data Availability

Not applicable.
